# Protein Removal from Hydrogels through Repetitive Surface Degradation

**DOI:** 10.1021/acsabm.1c00993

**Published:** 2021-11-19

**Authors:** Tatsuki Kamiya, Syuuhei Komatsu, Akihiko Kikuchi

**Affiliations:** Department of Materials Science and Technology, Tokyo University of Science, 6-3-1 Niijuku, Katsushika-ku, Tokyo 125-8585, Japan

**Keywords:** hydrogels, thermoresponsive, degradation, 2-methylene-1,3-dioxepane, hydrogel surface, protein removable surface

## Abstract

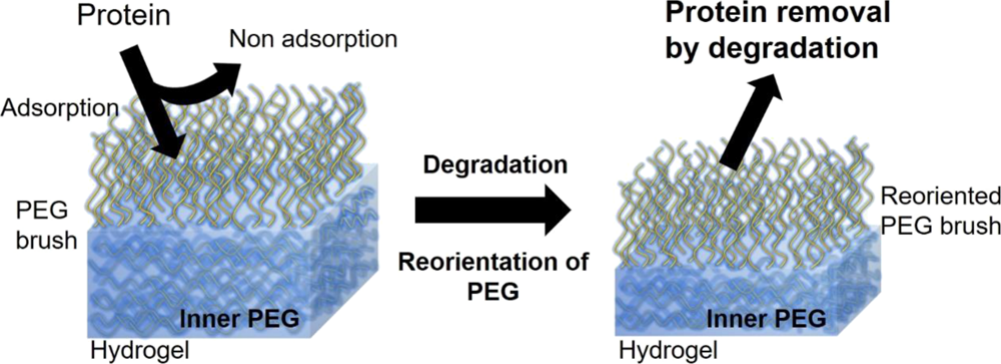

Suppression of protein adsorption is a necessary property for materials used in the living body. In this study, thermoresponsive and degradable hydrogels were prepared by the radical polymerization of 2-methylene-1,3-dioxepane, 2-hydroxyethyl acrylate (HEA), and poly(ethylene glycol) monomethacrylate (PEGMA). The prepared hydrogels re-exposed PEG-grafted chains to the interface through surface degradation, which was confirmed by the maintenance of the chemical composition of the hydrogel surfaces after hydrolysis. Notably, adsorbed proteins can be removed from the hydrogel surfaces through hydrogel surface degradation at least thrice.

## Introduction

Protein adsorption on material surfaces is triggered in living organisms as soon as biological fluids such as blood come in contact with the surfaces of artificial materials. Protein adsorption on material interfaces plays a key role in subsequent biological phenomena, such as cell adhesion^1^ and blood coagulation,^2^ and in biodevices used in biomedical applications. Suppression of the nonspecific adsorption of proteins is thus considered a crucial function to prevent biological reactions on biomaterial surfaces. To suppress protein adsorption on biomaterial surfaces, various hydrophilic polymers such as poly(ethylene glycol) (PEG),^3,4^ poly(*N*,*N*-dimethylacrylamide),^5^ hydrophilic zwitterionic polymers, poly(2-methacryloyloxyethyl phosphorylcholine) (MPC),^6^ sulfobetaine, and carboxybetaine polymers^7,8^ have been investigated, and some of them have already been applied to biomedical devices. These hydrophilic polymers show low-fouling properties owing to the formation of a hydration layer, which can effectively suppress the hydrophobic interactions of proteins with polymer surfaces. However, the majority of materials fail to obtain such properties. Removal of adsorbed proteins on the contact lens surfaces using lysozymes was reported for daily care of the lenses.^9^ Schulze et al. reported membrane surfaces that are able to self-clean adsorbed proteins using covalently immobilized enzymes.^10^ Surface cleaning and regeneration are important properties; however, it is difficult to facile design the surface that can be cleaned regardless of the type of protein. Moreover, there are possibilities such as enzyme inactivation and side reactions due to enzyme reactions. Degradable polymers with dynamic self-renewing surfaces have been reported as low-fouling marine materials.^11,12^ The degradable surfaces removed microorganisms such as diatoms and marine bacteria via surface renewal through degradation. These surfaces exhibited low-fouling properties for microorganisms and eventually prevented biofilm formation. Surface degradation can be achieved by introducing hydrophobic degradable groups. In addition, nonspecifically adsorbed proteins on the material surfaces can also be removed by degradation of the low-fouling surfaces. After degradation, the surfaces are renewed and they expressed the low-fouling property, repeatedly. The introduction of hydrophobic segments results in enhanced mechanical strength of materials and coating ability, but hydrophobicity often causes various drawbacks such as nonspecific protein adsorption and agglomeration of the degraded materials. Nevertheless, biomaterials must ideally maintain the functions of low protein adsorption and release of hydrophilic oligomers to be excreted from the body after degradation and surface renewal.

**Figure 1:**
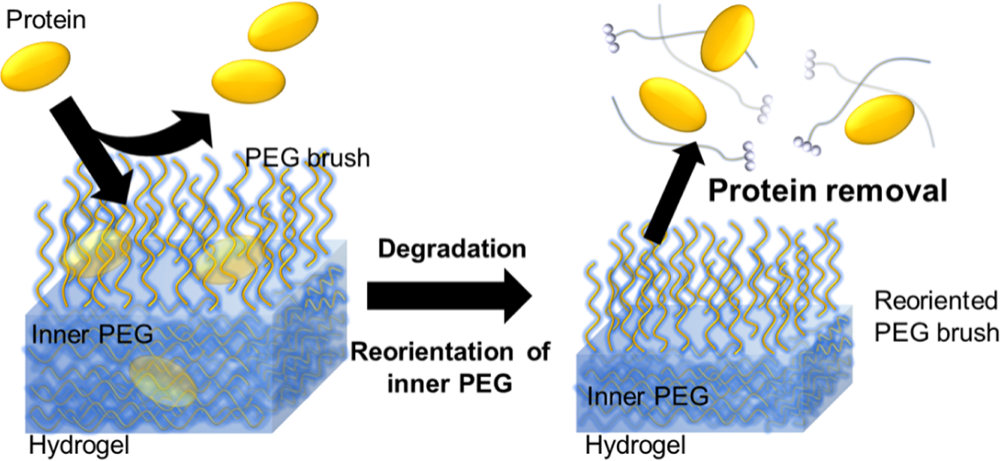
Illustration of protein removal by degradation of the hydrogel.

We hypothesized that degradable and hydrophilic hydrogel surfaces capable of surface renewal would exhibit low protein adsorption/release through surface degradation. We recently reported the preparation and characterization of degradable and thermoresponsive hydrogels by the radical copolymerization of 2-methylene-1,3-dioxepane (MDO) and 2-hydroxyethyl acrylate (HEA).^13^ The degradation of the prepared hydrogels could be controlled in terms of bulk degradation or surface degradation by the thermoresponsive swelling–deswelling property. Degraded HEA-based oligomers were soluble in water. We further reported PMDO-*g*-PEG nanoparticles,^15^ in which PEG chains remaining in the particle core were relocated and oriented to the surface of the particles after surface degradation, resulting in the maintenance of the dispersity of the particles. By combining these characteristics of PMDO-based polymers, it would be possible to design novel hydrogels with surface regeneration that maintain hydrophilic surfaces through the reorientation of PEG chains. The thermoresponsive behavior of the hydrogels could be utilized to control the degradation behavior of the hydrogel surfaces. This behavior would lead to the removal of adsorbed proteins and renewed interfaces of the hydrogels, with the PEG chains reoriented outward (Figure 1).

Herein, we report the synthesis of poly(MDO-*co*-HEA-*g*-PEG) hydrogels in the presence of a cross-linker. The hydrogels showed thermoresponsive and degradable properties with the reorientation of PEG chains on the surfaces of the hydrogels. Moreover, adsorbed proteins can be removed at least three times through surface degradation of the hydrogels.

## Experimental Section

### Materials

2,2′-Azobis(4-methoxy-2,4-dimethylvaleronitrile) (V-70), fluorescein-4-isothiocyanate, dimethyl sulfoxide (DMSO), *N*,*N*′-methylenebis(acrylamide) (MBAAm), and HEA were purchased from FUJIFILM Wako Pure Chemical Corporation. (Osaka, Japan). DMSO was distilled under reduced pressure before use (0.5 kPa, 95.0 °C). Poly(ethylene glycol) monomethacrylate (PEGMA) (*M*_w_ 2000), bovine serum albumin-fluorescein isothiocyanate conjugate (FITC–BSA), and fibrinogen fraction I type I-S were purchased from Sigma-Aldrich (MO, USA). 2-Methylene-1,3-dioxepane (MDO) was prepared by a two-step reaction according to the previous reports.^13−16^ FITC–fibrinogen was synthesized from fibrinogen and FITC in carbonate buffer (pH 8.5) for 20 h at 25 °C.

### Preparation of Poly(MDO-*co*-HEA-*g*-PEG) Hydrogels

**Scheme 1:**
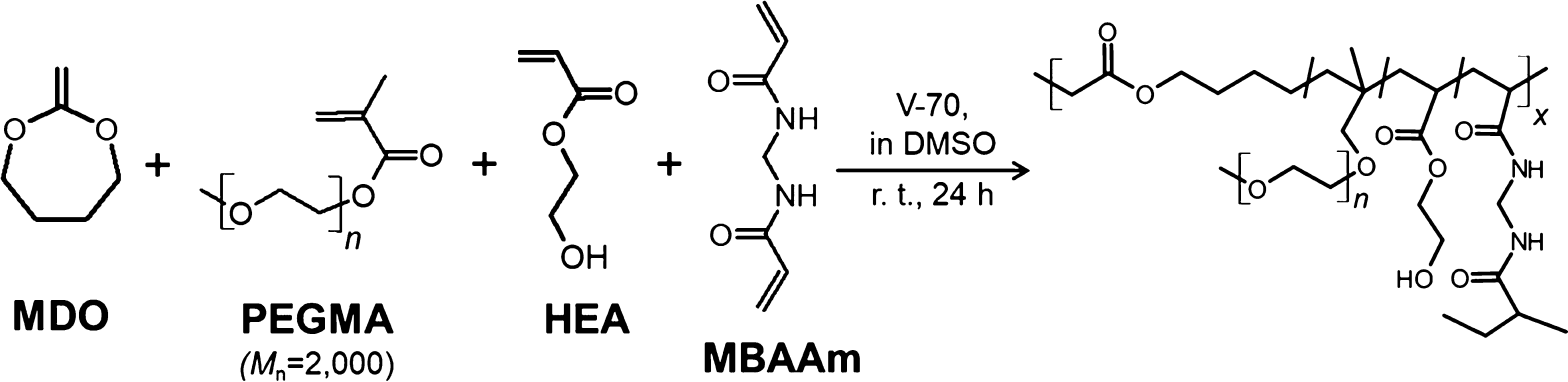
Synthesis of Thermoresponsive Degradable Hydrogels via Radical Polymerization

Poly(MDO-*co*-HEA-*g*-PEG) hydrogels were prepared by radical polymerization of MDO, HEA, and PEGMA in the presence of MBAAm as a cross-linker and V-70 (2 mol % to monomer) as a radical initiator in DMSO (Scheme 1), according to previous reports.^13,15^ Briefly, pre-gel solution degassed by bubbling N_2_ gas for # min was injected between two glass plates sandwiching a 0.5 mm-thick poly(dimethylsiloxane) spacer. Gelation was carried out for 24 h at 25 °C, after which these hydrogels were purified in methanol and ultrapure water to remove unreacted monomers and cross-linker. After purification, the poly(MDO-*co*-HEA-*g*-PEG) hydrogels were cut into disk (diameter 0.9 cm and thickness 0.5 mm).

### Thermoresponsive Behavior of Poly(MDO-*co*-HEA-*g*-PEG) Hydrogels

Thermoresponsive behavior of the prepared hydrogels was evaluated by measuring the change in the swelling ratio. The prepared hydrogel disc was immersed in ultrapure water for 24 h at 45 °C to reach equilibrium swelling. After that, the temperature changed to predetermined temperature (5–45 °C), and the hydrogels were incubated for 48 h to attain equilibrium swelling at each temperature. The swelling ratio of the hydrogels was calculated from the weight of the swollen gels (*W*_s_) and that of the dry gels (*W*_d_) using the following equation
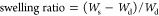
1

### Degradation Behavior of Poly(MDO-*co*-HEA-*g*-PEG) Hydrogels

Degradation of the hydrogels was determined by alkaline hydrolysis in 1.0 mmol/L NaOH aq. as an accelerated test at either 10 or 37 °C. The degradation behavior was estimated by means of the change in the swelling ratio, ^1^H NMR measurement of supernatant solution using AVANCE Neo 400 (Bruker, USA), and ATR FT-IR measurement of the dried hydrogel surfaces using FT/IR-4200 equipped with the ATR unit using Ge crystals (JASCO, Tokyo) at an integration number of 64, respectively.

### Repetitive Protein Removal Property of the Poly(MDO-*co*-HEA-*g*-PEG) Hydrogel

Protein removal behavior was determined by the change in the fluorescence intensity of hydrogel surfaces before and after degradation. The poly(MDO-*co*-HEA-*g*-PEG) hydrogels were incubated in ultrapure water for 24 h at 37 °C to equilibrium swelling. The hydrogel discs were then soaked in FITC–BSA or FITC–fibrinogen solution (0.1 mg/mL in PBS, pH 7.4) at 37 °C for 1 h. The surface of the hydrogel disc was gently washed with ultrapure water, and excess water was removed with Bemcot, followed by soaking in NaOH solution (1.0 mmol/L) for 1 h at 37 °C. The hydrogel was washed again with ultrapure water. The fluorescence image of the degraded hydrogel was observed and recorded using a fluorescence microscope (BZ-8100, Keyence, Osaka). This method was repeated three times to evaluate the protein removal properties. The signal-to-blank ratio was defined as the difference in the fluorescence intensity of protein on the hydrogels (*F*_h_) and fluorescence intensity of bare glass (*F*_g_), calculated using Image J software ver. 1.51 (National Institute of Health, USA) using the following equation
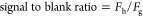
2

## Results and Discussion

Hydrogels were synthesized via the free-radical copolymerization of the corresponding monomer mixture in the presence of a cross-linker in DMSO (Scheme 1). The temperature-dependent changes in the swelling ratios of the hydrogels were examined in ultrapure water. The swelling ratio of the hydrogels decreased from 10.5 at 10 °C to 3.9 at 40 °C (Figure S1), indicating that the hydrogels showed shrinking behavior with temperature. The balance of hydrophilic (HEA and PEG) and hydrophobic (MDO) segments affects the expression of thermoresponsive properties.^13,15,16^ An increase in the feed composition of MDO induced lower swelling ratios in all temperature ranges examined (Figure S1).

**Figure 2:**
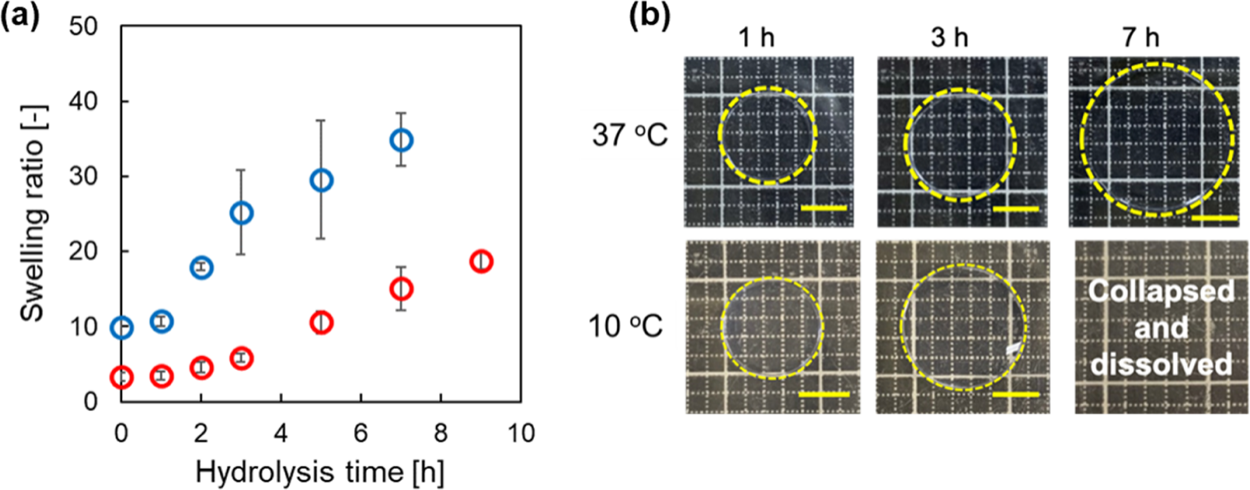
Characterization of hydrogels during alkaline hydrolysis. Hydrogels prepared with the composition of (MDO + HEA)/PEGMA = 100:1 (mol/mol) and MDO/HEA = 6:4 (mol/mol). (a) Hydrolysis time-dependent changes in swelling ratios of the hydrogels at 37 and 10 °C. Data are expressed as the mean ± SD (*n* = 3). Red plot: 37 °C and blue plot: 10 °C. (b) Optical images of the hydrogel during hydrolysis at 10 and 37 °C.

Next, we examined the degradation behavior of the prepared hydrogels. The degradability of the hydrogels was investigated under alkaline conditions (pH 11.3) as an accelerated test. Figure 2a shows the hydrolysis time-dependent change in the swelling ratio for the hydrogels made with the composition MDO/HEA = 6:4 (mol/mol) in 1.0 mmol/L NaOH solution (pH 11.3) at 10 and 37 °C. The swelling ratio of the hydrogels increased with increasing hydrolysis time, regardless of the temperature. The tested hydrogels showed a gradual increase in the swelling ratio and complete degradation and dissolution after 10 h of incubation in 1.0 mmol/L NaOH solution at 37 °C. Furthermore, at 10 °C, a substantially greater increase in the swelling ratio was observed, and after 7 h, the hydrogels were completely degraded. Therefore, the swollen hydrogels are susceptible to degradation. The swelling ratio of the hydrogels prepared at MDO/HEA = 7:3 (mol/mol) showed similar trends during hydrolysis, but the swelling ratios were larger than those of the hydrogels prepared at MDO/HEA = 6:4 (mol/mol) (Figure S2). This is probably due to the difference in the number of ester groups that are susceptible to hydrolysis in the hydrogels. The slow degradation behavior of the hydrogels at 37 °C suggested that surface degradation occurred initially in the shrunken hydrogels rather than degradation deep inside the hydrogels. By contrast, the swollen hydrogels were hydrolyzed not only at the surface but also in the bulk at 10 °C; thus, a sharp increase in the swelling ratio was observed within a short time period (Figure 2a,b). These results indicated that the hydrogels showed degradable properties, and the ester groups derived from MDO in the polymer backbone were cleaved by hydrolysis. It was also indicated that the degradation behavior of the hydrogels could be controlled by temperature.

**Figure 3:**
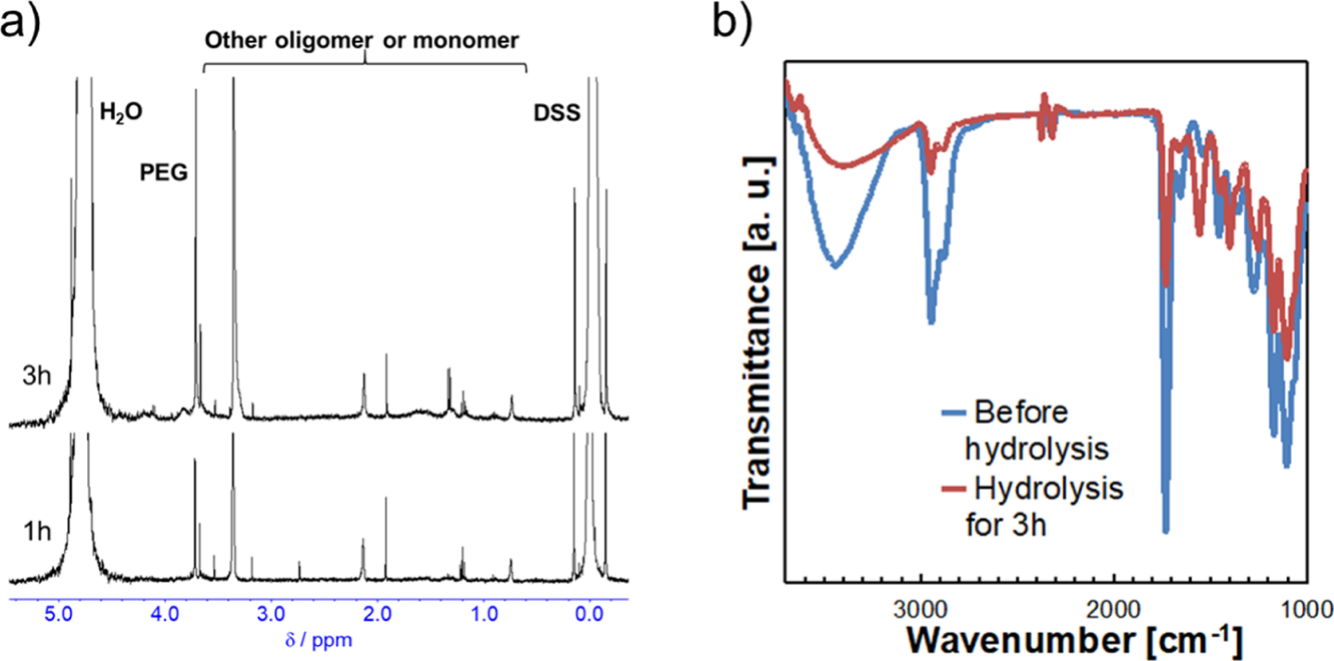
Characterization of the hydrogel surface and degradation supernatant during alkaline hydrolysis. Hydrogels were prepared with the composition of (MDO + HEA)/PEGMA = 100:1 (mol/mol) and MDO/HEA = 6:4 (mol/mol). (a) ^1^H NMR spectra of supernatants during hydrolysis of hydrogels for 1 and 3 h. (b) ATR-FTIR spectra of dried hydrogels before and after hydrolysis for 3 h.

We then investigated the hydrogel surfaces before and after degradation to determine the surface functionalities of the hydrogels by ^1^H NMR and ATR-FTIR measurements. The ^1^H NMR spectra of the supernatant solutions were measured during the hydrolysis of the hydrogels to determine their degradation behavior. Figure 3a shows the ^1^H NMR spectra of the supernatant solution of the hydrogels after 1 and 3 h of hydrolysis in D_2_O containing 1.0 mmol/L NaOD. A peak corresponding to PEG appeared at 3.8 ppm in the ^1^H NMR spectrum for the supernatant solution in the case of 1 h hydrolysis, and the peak intensity increased after 3 h hydrolysis. This result indicates that the PEG chains cleaved by hydrolysis were released into the solution, and thus, the peaks corresponding to PEG intensified with hydrolysis time. The surface of the hydrogel during hydrolysis was evaluated by ATR-FTIR (Figure 3b). The native hydrogels and the hydrogels immersed in 1.0 mmol/L NaOH solution for 3 h were freeze-dried and measured on germanium crystals. Before hydrolysis, the peaks of ether, ester, and hydroxy groups derived from the corresponding monomer units in the hydrogels were confirmed at 1150, 1750, and 3200–3600 cm^–1^, respectively. These three peaks were observed even after partial degradation of the hydrogels, although the signal intensities decreased. These results suggest that the hydrogels showed similar surface compositions before and after hydrolysis, showing that the hydrogels exhibit surface renewability through the re-orientation of the PEG chains on their surfaces.

**Figure 4:**
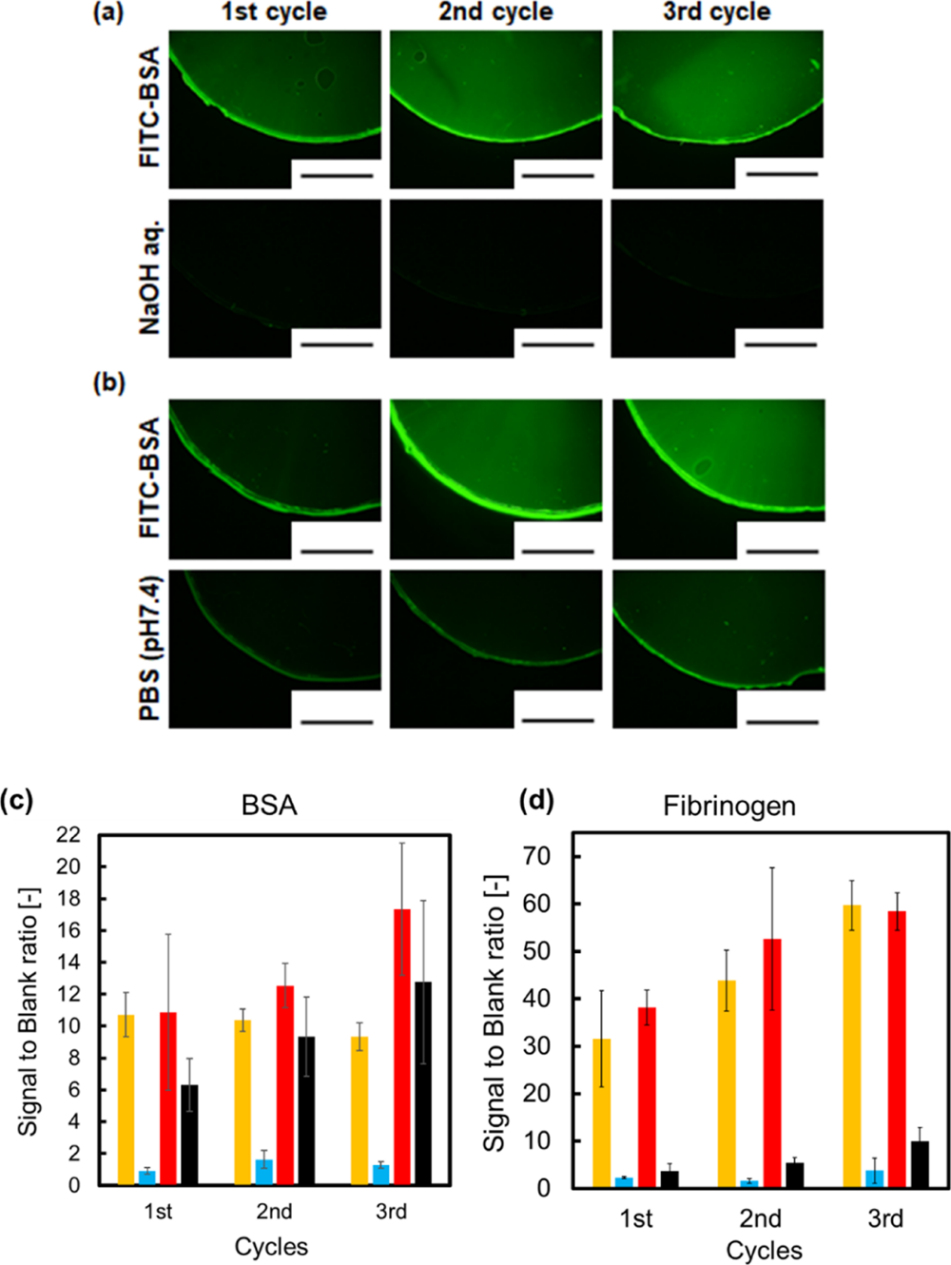
Fluorescence microscopic images of hydrogels (a) immersed in FITC–BSA solution (1.0 mg/mL in PBS, pH 7.4) for 1 h (upper) and hydrolyzed in 1.0 mmol/L NaOH solution for 1 h (bottom) and (b) immersed in FITC–BSA solution (1.0 mg/mL in PBS, pH 7.4) for 1 h (upper) and PBS for 1 h (bottom). (c) Signal intensity ratio of adsorbed BSA on hydrogels. Yellow and red bars: the signal intensity ratio for the hydrogels immersed in FITC–BSA solution for 1 h. Blue bars: the signal intensity ratio for the hydrogels immersed in 1.0 mmol/L NaOH solution for 1 h after 1 h incubation in FITC–BSA solution. Black bars: The signal intensity ratio for the hydrogels immersed in PBS for 1 h after 1 h incubation in FITC–BSA solution. (d) Signal intensity ratio of adsorbed fibrinogen on hydrogels. Yellow and red bars: the signal intensity ratio for the hydrogels immersed in fibrinogen solution for 1 h. Blue bars: the signal intensity ratio for the hydrogels immersed in 1.0 mmol/L NaOH solution for 1 h after 1 h incubation in fibrinogen solution. Black bars: the signal intensity ratio for the hydrogels immersed in PBS for 1 h after 1 h incubation in fibrinogen solution.

Finally, the protein adsorption and surface renewal properties of the hydrogels were investigated by fluorescence microscopy. We investigated the surface renewal properties by repetitive tests of protein adsorption and removal. The hydrogels were immersed in either fluorescein-isothiocyanate-labeled bovine serum albumin (FITC–BSA) or FITC-labeled fibrinogen solution (0.5 mg/mL in PBS, pH7.4, 37 °C) for 1 h, followed by gentle washing in PBS. Subsequently, the hydrogels were immersed in 1.0 mmol/L NaOH solution or PBS for 1 h at 37 °C. Fluorescence microscopic observations and fluorescence measurements were conducted at each step over three cycles (Figure 4a,b). In the first cycle, fluorescence derived from FITC–BSA was observed on both hydrogels (Figure 4a). Immersed in PBS at 10 °C, the fluorescence intensity of the hydrogel was increased compared with that immersed at 37 °C (Figure S3). Because the hydrogel showed expansion of the gel network (Figure S1) and protein diffusion inner hydrogel at low temperature due to thermoresponsive property, it is suggested that the BSA was adsorbed near the surface at 37 °C due to decreased fluorescence intensity. After hydrolysis, the fluorescence on the hydrogels disappeared, whereas the fluorescence intensity decreased but did not disappear for the hydrogels immersed in PBS (Figure 4b). The same trends were observed for the second and third cycles. The amounts of adsorbed proteins, BSA, and fibrinogen were compared based on the signal-to-blank ratio of the fluorescence intensity of the hydrogels (Figure 4c,d). The adsorbed amount of BSA was in the range of 60–100 ng/cm^2^, which suggested the monolayer adsorption of BSA on the hydrogel surfaces. This may be due to the relatively weak interaction of BSA with the PEG-tethered hydrogel surfaces. The signal-to-blank ratio of the hydrogels immersed in PBS for washing increased with an increase in the number of cycles. Furthermore, the fluorescence intensity of the hydrogels subjected to alkaline hydrolysis decreased to the equivalent values for the blank. Moreover, the same tendency was observed for fibrinogen adsorption, whereby the fluorescence intensity of the hydrogels decreased to equivalent values for the blank by alkaline hydrolysis. BSA adsorption at 37 °C was in the range of monolayer adsorption, while a more amount of fibrinogen may be adsorbed on hydrogel surfaces. After rinsing with PBS, the signal intensity of the remaining BSA on the hydrogel was slightly higher than that of the remaining fibrinogen on the hydrogel (black bars in Figure 4c,d). The extent of fibrinogen removal looked larger than that of BSA, but it cannot be compared because the modification degree of fluorescent molecules per protein was different. The other reason may be the difference in the size of proteins, BSA, and fibrinogen. The size of BSA is 4 × 4 × 14 nm,^17^ while that of fibrinogen is 45 × 9 × 7 nm.^18^ The smaller size protein, BSA, may have higher occupancy on the hydrogel surfaces than fibrinogen on the basis of molecular sizes. These results indicate that surface degradation was effective for the removal of adsorbed proteins. Therefore, the prepared hydrogels repetitively exhibit surface renewal properties by the degradation of hydrogels at least three times.

## Conclusions

In conclusion, thermoresponsive and degradable hydrogels with re-orientation of PEG chains on the surface of hydrogels were synthesized. The prepared hydrogels showed re-orientation property of PEG chains and regenerating low-fouling property by surface degradation of the hydrogels. The prepared hydrogels would be expected as novel biomaterials exhibiting regenerating low-fouling property.

## Supporting Information Available

Temperature-dependent swelling ratios for hydrogels with different compositions of the hydrogel (MDO/HEA = 6:4 and 7:3); degradable properties of hydrogels by alkaline hydrolysis as an accelerated test (1.0 mmol L1 NaOH) at 10 and 37 °C; and fluorescence microscopic images of hydrogels (monomer/PEG = 100:1) immersed in FITC–BSA solution (1.0 mg/mL in PBS, pH 7.4) for 1 h at 10 and 37 °C (PDF)
