# Supplementary material for: Protein Removal from Hydrogels through Repetitive Surface Degradation
Source: ACS Appl Bio Mater. 2021 Nov 19;4(12):8498–502. doi: 10.1021/acsabm.1c00993 (PMC8693177; doi:10.1021/acsabm.1c00993)
Supplement: Supplementary file 1 — mt1c00993_si_001.pdf [file mt1c00993_si_001.pdf]

Supporting Information

Protein removal from hydrogels through repetitive surface degradation

*Tatsuki Kamiya, Syuuhei Komatsu, Akihiko Kikuchi**

[1] Department of Materials Science and Technology, Tokyo University of Science, 6-3-1 Niijuku, Katsushika-ku, Tokyo 125-8585, Japan

* Corresponding author: Akihiko Kikuchi
Phone: +81-3-5876-1415; Fax: +81-3-5876-1639; E-mail: kikuchia@rs.tus.ac.jp

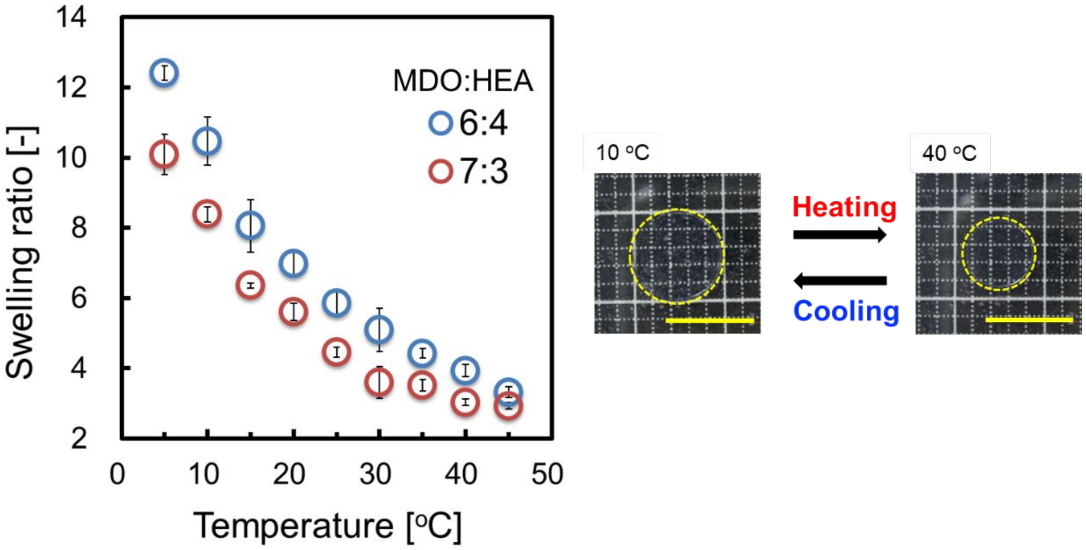

Figure S1 Temperature-dependent swelling ratios for hydrogels with different compositions. Blue plot: MDO:HEA=6:4 (mol/mol), MDO+HEA:PEG=100:1 (mol/mol), Red plot: MDO:HEA=7:3 (mol/mol), MDO+HEA:PEG=100:1 (mol/mol).

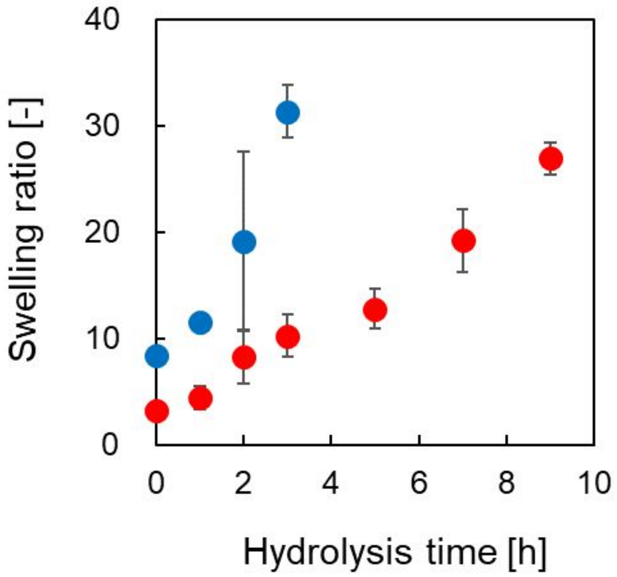

Figure S2 Degradable properties of hydrogels by alkaline hydrolysis as an accelerated test (1.0 mmol L^{-1} NaOH) at 10 °C and 37°C. Change in swelling ratio of the hydrogels. Blue plot: MDO: HEA=7:3 (mol/mol), MDO+HEA: PEG=100:1 (mol/mol) at 10°C, Red plot: MDO: HEA=7:3 (mol/mol), MDO+HEA: PEG=100:1 (mol/mol) at 37°C. Data are expressed as the mean with standard deviation of triplicate experiments.

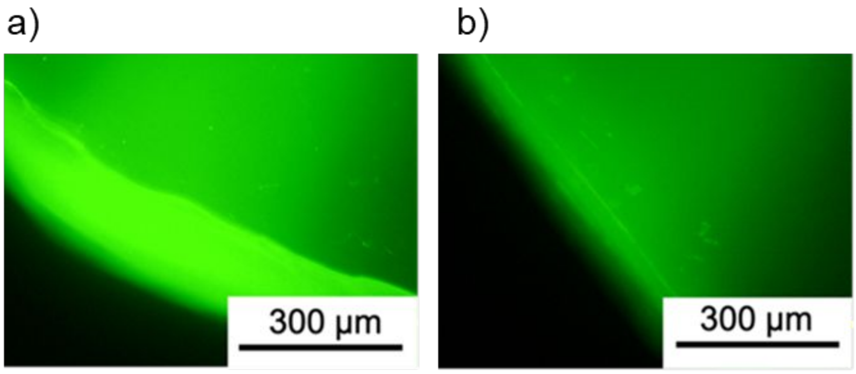

Figure S3 Fluorescence microscopic images of hydrogels (Monomer: PEG=100:1) immersed in FITC-BSA solution (1.0 mg/mL in PBS, pH 7.4) for 1h at a) 10°C and b) 37°C.
